# Supplementary material for: Seminoma and Embryonal Carcinoma Footprints Identified by Analysis of Integrated Genome-Wide Epigenetic and Expression Profiles of Germ Cell Cancer Cell Lines
Source: PLoS One. 2014 Jun 2;9(6):e98330. doi: 10.1371/journal.pone.0098330 (PMC4041891; doi:10.1371/journal.pone.0098330)
Supplement: File S1 — ZIP file containing DMRforPairs output for significant regions. Please start from the html files. (ZIP) [file pone.0098330.s008.zip › figures/27905.pdf]

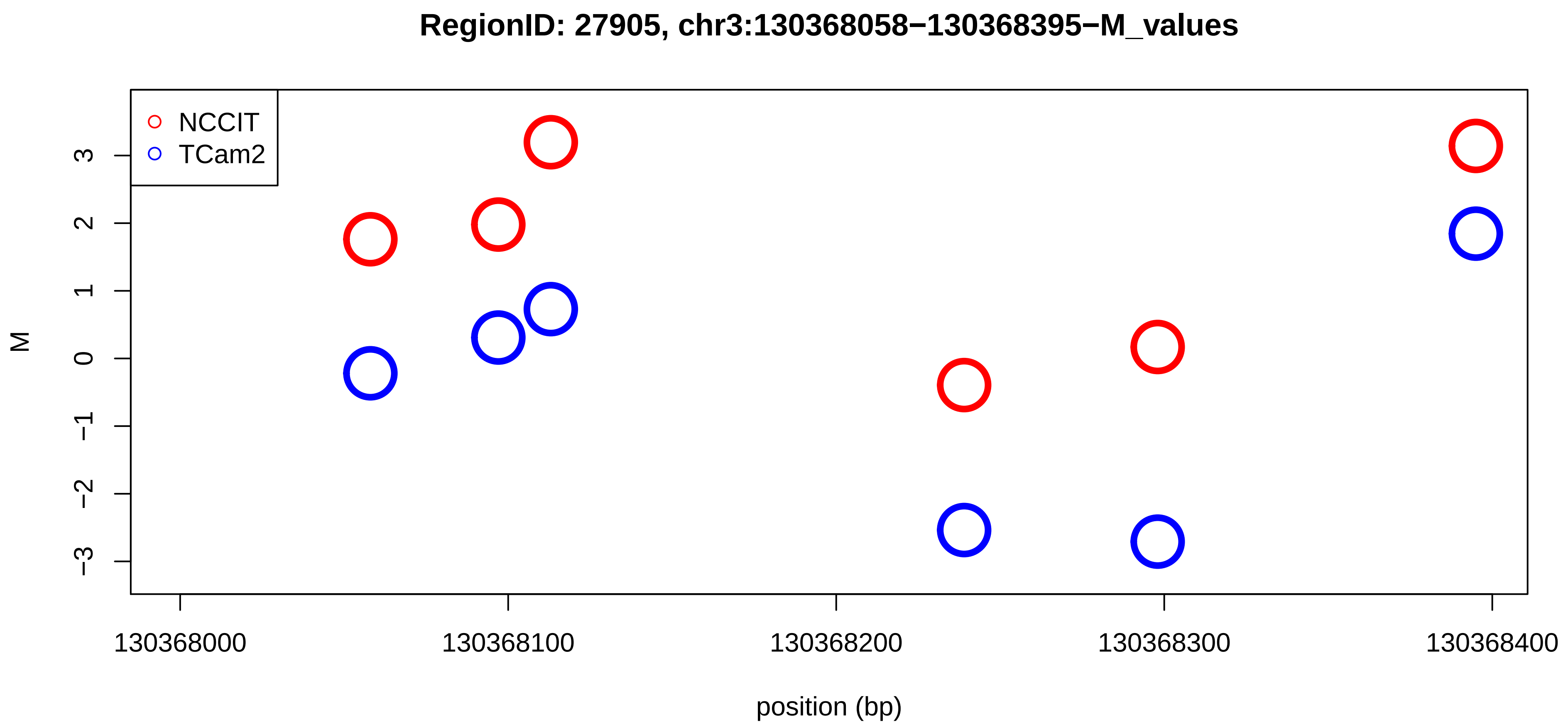
RegionID: 27905, chr3:130368058−130368395−M_values
NCCIT
TCam2
M
−3
−2
−1
0
1
2
3
130368000
130368100
130368200
130368300
130368400
position (bp)

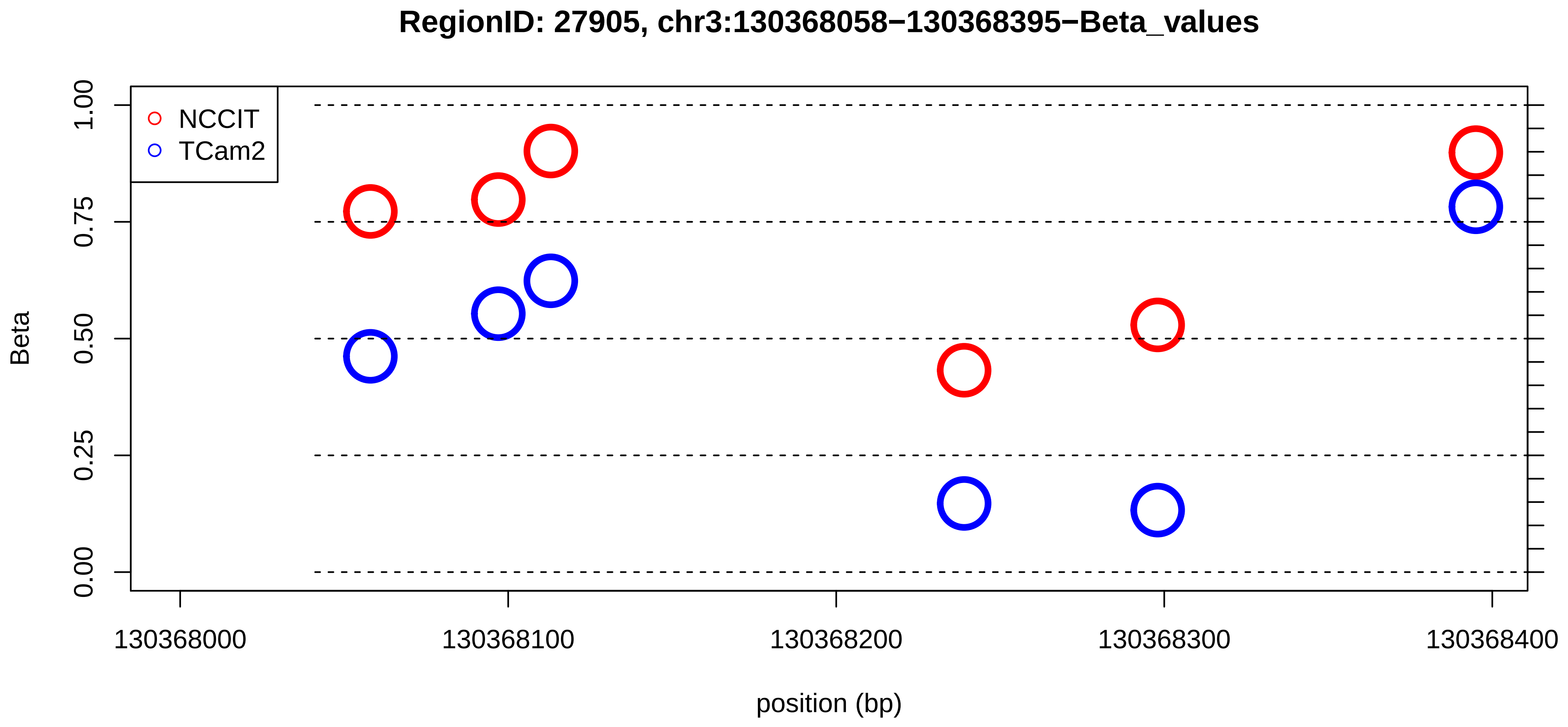
RegionID: 27905, chr3:130368058−130368395−Beta_values
NCCIT
TCam2
Beta
0.00
0.25
0.50
0.75
1.00
130368000
130368100
130368200
130368300
130368400
position (bp)
